# Understanding professional advice networks in long-term care: an outside-inside view of best practice pathways for diffusion

**DOI:** 10.1186/s13012-019-0858-6

**Published:** 2019-01-30

**Authors:** Lisa A. Cranley, Janice M. Keefe, Deanne Taylor, Genevieve Thompson, Amanda M. Beacom, Janet E. Squires, Carole A. Estabrooks, James W. Dearing, Peter G. Norton, Whitney B. Berta

**Affiliations:** 1 Lawrence S. Bloomberg Faculty of Nursing, University of Toronto, Toronto, Ontario Canada; 2 Nova Scotia Centre on Aging and Department of Family Studies and Gerontology, Mount Saint Vincent University, Halifax, Nova Scotia Canada; 3 Research Department, Interior Health Authority, Kelowna British Columbia and School of Nursing, Faculty of Health and Social Development, University of British Columbia (Okanagan campus), Kelowna, BC Canada; 4 Rady Faculty of Health Sciences, College of Nursing, University of Manitoba, Winnipeg, Manitoba Canada; 5 Carroll School of Management, Boston College, Chestnut Hill, MA USA; 6 Ottawa Hospital Research Institute and Faculty of Health Sciences, School of Nursing, University of Ottawa, Ottawa, Ontario Canada; 7 Faculty of Nursing, University of Alberta, Edmonton Clinic Health Academy, Edmonton, Alberta Canada; 8 Department of Communication, Michigan State University, East Lansing, MI USA; 9 Department of Family Medicine, University of Calgary, Calgary, Alberta Canada; 10 Dalla Lana School of Public Health, Institute of Health Policy, Management and Evaluation, University of Toronto, Toronto, Ontario Canada

**Keywords:** Diffusion of innovations, Long-term care sector, Mixed methods, Professional advice seeking networks

## Abstract

**Background:**

Interpersonal relationships among professionals drive both the adoption and rejection of consequential innovations. Through relationships, decision-makers learn which colleagues are choosing to adopt innovations, and why. The purpose of our study was to understand how and why long-term care (LTC) leaders in a pan-Canadian interpersonal network provide and seek advice about care improvement innovations, for the eventual dissemination and implementation of these innovations.

**Methods:**

We used a mixed methods approach. An online survey was sent to senior leaders in 958 LTC facilities in 11 Canadian provinces and territories. Participants were asked to name up to three individuals whose advice they most value when considering care improvement and practice innovations. Sociometric analysis revealed the structure of provincial-level advice networks and how those networks were linked. Using sociometric indicators, we purposively selected 39 key network actors to interview to explore the nature of advice relationships. Data were analyzed thematically.

**Results:**

In this paper, we report our qualitative findings. We identified four themes from the data. One theme related to characteristics of particular network roles: opinion leaders, advice seekers, and boundary spanners. Opinion leaders and boundary spanners have long tenures in LTC, a broad knowledge of the network, and share an interest in advancing the sector. Advice seekers were similarly committed to LTC; they initially seek and then, over time, exchange advice with opinion leaders and become an important source of information for them. A second theme related to characterizing advice seeking relationships as formal, peer-to-peer, mentoring, or reciprocal. The third and fourth themes described motivations for providing and seeking advice, and the nature of advice given and sought. Advice seekers initially sought information to resolve clinical care problems; however, over time, the nature of advice sought expanded to include operational and strategic queries. Opinion leaders sought to expand their networks and to solicit information from their more established advice seekers that might benefit the network and advance LTC.

**Conclusions:**

New knowledge about the distinct roles that different network actors play vis-a-vis one another offers healthcare professionals, researchers, and decision- and policy-makers insights that are useful when formulating best practice dissemination strategies.

The diffusion of innovations relies on the communication of both descriptive and evaluative information among people [1, 2]. One tenet of innovation diffusion theory is that while descriptive information is mostly accessed via impersonal channels of communication such as websites and specialty media, evaluative information is primarily exchanged interpersonally, from person to person, as advice or example [1]. Knowledge about innovations in healthcare is more likely to be gleaned from members of professional social networks than from the information that is available through academic journals, published guidelines, and conference presentations [3–6]. Social network analysis has been used to understand how advice about innovations moves through a network and to develop “network interventions” [7]. Such interventions can enhance the efficiency of innovation dissemination by altering the network structure [8, 9] or expedite a desired behavioral change [7, 10, 11]—like increasing the uptake of an evidence-based practice—by tapping into the existing network structure.

Opinion leadership and boundary spanning are key roles within a professional social network. Opinion leaders, because of their credibility in terms of expertise, trustworthiness, and accessibility, are central to communication structures and information flow [12]. The reasons for conferral of informal opinion leader status can vary by setting and innovation [1, 8]. Boundary spanners move knowledge and information from group to group across a network, thus helping to pollinate groups with new ideas for opinion leaders to consider and playing the heterogenous function of communicating innovations across networks by bridging network gaps (e.g., structural holes) [13, 14].

Quantitative approaches to social network analysis afford an “outsider view” of networks, mapping and measuring “aspects of social relations in a systematic and precise fashion” ([15], p.5). Qualitative approaches are less common and afford an “insider view,” exploring the subjective meaning of a network to members and elucidating the reasons for individual behavior [16]. In mixed methods studies of social networks, these complementary approaches are brought together to offer a particularly rich “outside-inside view” of social relations and a nuanced understanding of the structure of the network and the forces that produce it [16, 17].

In Canada, long-term care (LTC) facilities are governed by each province or territory. Many jurisdictions delegate operations oversight, including regulatory compliance, to regional health authorities [18]. These deliver public healthcare, and their boards are accountable to the provincial health ministry [19]. LTC facilities may be public not-for-profit, voluntary (e.g., faith-based) not-for-profit, or private (for-profit or not-for-profit) [20]. The fragmented governance of the Canadian LTC system likely increases the “stickiness” [21] of best practices and decreases cross-jurisdictional diffusion. As with the USA, the care needs of residents in the Canadian LTC system are becoming increasingly complex resulting from multi-morbidities [22, 23]. Facility leaders are increasingly challenged to find innovative ways to respond to these needs and sustain acceptable levels of care quality [24]. While one source of practice innovations lies in the experiences and knowledge of other facility leaders, and other professionals, in the LTC system in Canada, the fragmented governance of the system appears to constrain the development of a porous system-wide network and decreases the likelihood of cross-jurisdictional diffusion [20].

We conducted a two-phased study using a mixed methods approach to understand the relations and interactional processes of a pan-Canadian advice seeking network of professionals in the residential LTC sector. Our chief motivation was to inform future cross-jurisdictional LTC best practice dissemination efforts through insights about extant advice seeking among LTC directors, i.e., through identification of “pathways” for diffusion to occur. In the first phase of the study, we examined advice networks both within and across 11 Canadian provinces and territories in Atlantic and Western and Northern Canada. We completed a formal, quantitative social network analysis of the advice seeking networks among senior LTC leaders working in these jurisdictions; these results are published in a prior issue of this journal [20]. In the second phase of the study, we aimed to better understand the nature of the advice relationships, the characteristics of those who hold key roles in the network—opinion leaders, boundary spanners, and advice seekers—and the types of advice sought and given. The present article focusses on findings from this second, qualitative phase of our study.

## Methods

### Participant selection

Our qualitative sample selection was informed from our social network survey data analysis. In the quantitative phase, we sent an online survey to one senior leader (e.g., Director of Care, Director of Nursing) in each of the 958 LTC facilities operating across eight provinces and three territories in Atlantic and Western and Northern Canada. Because survey response rates were low (< 30%) in Newfoundland and Labrador, Yukon, and Nunavut, we excluded those provinces and territories from the interview sampling plan. In the survey phase of the study, we asked: “Whose advice do you most value about delivery of quality care, care improvement and innovation?” We approached senior leaders because they are best positioned to seek and implement knowledge and advice about care delivery in LTC. Participants were asked to name up to three individuals external to their facility and in the order of their importance. Named individuals were not restricted to peers working in other facilities, and individuals in government, corporate management, consulting, and academic research were named. We then generated sociograms using the survey data; these were maps of relationships in the interpersonal advice network of each province or territory accompanied by metrics measuring the network position and role of each member [20].

**Fig. 1:**
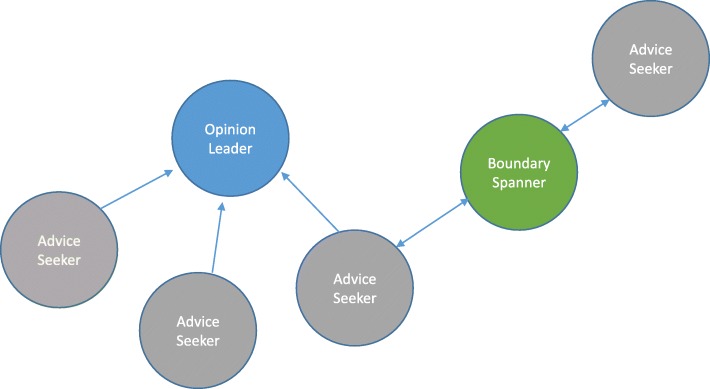
Simple professional advice network diagram. Opinion leader = had high in-degree centrality scores (number of incoming ties from others in the network). Boundary spanner = had high betweenness centrality scores and had at least one outgoing tie and one incoming tie from others. Advice seekers = had at least one outgoing tie

We used this survey data analysis to purposively identify three types of potential interview participants based on network position and role. *Opinion leaders* serve as sources of advice and of examples of how they responded to a given innovation and were selected by identifying the actors in each provincial/territorial network with the highest in-degree centrality scores (number of incoming ties from others in the network). *Boundary spanners* connect sociometrically distinct groups in the network and were selected by identifying the actors in each network with the highest betweenness centrality scores (the frequency with which an actor is positioned on the shortest path between other actors in the network) [25]. Boundary spanners who connected people from different provinces or people with different professional roles (e.g., senior leaders in LTC facilities vs. in regional and provincial governments) were prioritized as interview participants, because we were interested in insights on how barriers presented by fragmented governance, and professional silos, are overcome by such individuals.^1^ *Advice seekers* were defined as actors in each provincial network who had not already been selected as opinion leaders and boundary spanners and who sought advice from at least one opinion leader or boundary spanner.^2^ From our survey, there were 1140 members of the pan-Canadian interpersonal advice network, of whom 462 were advice seekers, 50 opinion leaders, and 51 boundary spanners [20] (note that these role-types were not exclusive, such that one member of the network could be an advice seeker, opinion leader, and boundary spanner simultaneously, and all boundary spanners were by definition also advice seekers). Using this initial sampling frame, we then began contacting potential interview participants from each category of network role in each province and territory (Fig. 1).

We completed 39 interviews: 13 with opinion leaders (3 Atlantic, 10 West/North), 22 with advice seekers (6 Atlantic, 16 West/North), and 4 with boundary spanners (2 Atlantic, 2 West/North) (Table 1). Interviews lasted on average 38 min (range, 18–74 min). All 39 participants were similar in age, 38 of 39 were female, all indicated English as their first language, and most had nursing backgrounds. Mean sample characteristics are similar to those reported from the online survey respondents [20]. All advice seekers and boundary spanners held senior leadership positions in LTC facilities. Ten opinion leaders held positions in government. Boundary spanners had the longest tenure in LTC (20 years on average) and in their current job (8 years on average) (Table 2).

**Table 1:** Summary of sampling frame by role

SNA role	Members of network identified from survey data	Number of potential participants in interview sample	Number of completed interviews
Advice seeker	462	69	22
Opinion leader	50	32	13
Boundary spanner	51	40	4

**Table 2:** Demographic characteristics of interview participants

	Advice seekers	Boundary spanners	Opinion leaders	Total
Professional role				
Senior leadership position in an LTC facility	22	4	1	27
Corporate-level position in LTC organization	0	0	2	2
Position in regional health authority/government	0	0	10	10
Works at > 1 facility^1^	3	2	0	5
Owner-operator model of facility^1^				
Public not-for-profit	5	2	0	7
Private for-profit	5	0	0	5
Voluntary not-for-profit	9	2	0	11
Private not-for-profit	2	0	1	3
Missing	1	0	0	1
Number of beds in facility^1^				
0–79	8	0	0	8
80–120	7	1	0	8
> 120	6	3	1	10
Missing	1	0	0	1
Gender				
Women	21	4	13	38
Men	1	0	0	1
Age				
20–39	1	0	1	2
40–59	20	1	11	32
60 +	1	3	1	5
First language				
English	22	4	13	39
Education				
Diploma/certificate	9	3	1	13
Bachelors	8	0	6	14
Graduate	5	1	6	12
Professional background				
Nursing	18	4	12	34
Business	2	0	1	3
Other	2	0	0	2
Tenure in LTC [M (SD)]	15.52 (9.98)	20.00 (10.68)	14.54 (10.48)	15.66 (10.06)
Tenure in current job [M (SD)]	6.82 (5.40)	8.25 (4.43)	5.31 (3.47)	6.46 (4.72)

Source authors’ analysis, *LTC* (long-term care)

^1^Applicable to those (*n* = 27) working in senior leadership positions in LTC facilities only

### Data collection

Qualitative data were collected using a semi-structured interview guide informed by Rogers’ work on roles of social ties and communication channels in innovation diffusion [1]. The interview guide was piloted with three Atlantic advice seekers to test the questions and language used. Pilot interview data were not transcribed or analyzed. Data were collected via telephone interviews conducted between fall 2015 and spring 2016 by at least one researcher and one student trainee. Participants were asked to describe their relationships and types of advice sought and how these changed over time; their motivations for entering into and sustaining relationships; and characteristics of individuals from whom they sought advice (e.g., advice seekers were asked to describe characteristics of opinion leaders and/or boundary spanners). Interviews were audio-recorded and transcribed verbatim, and informed consent was obtained before data collection.

### Data analysis

Data were analyzed using thematic analysis [26]. Data analysis was concurrent and iterative with data collection [27]. Transcripts were assigned to two researchers who first read them individually to become familiar with the data and to capture initial analytic thoughts and ideas. Next, two individuals systematically coded each transcript to identify and describe phenomena found in the text, line by line. We then met as a team and each team member presented their perspectives as part of the consensus coding process across a subset of transcripts. This approach was repeated until all transcripts were coded. Concurrently, codes were collated into categories and grouped into themes. While our interview guide was informed by Rogers’ work on innovation diffusion theory [1], specifically social ties and communication channels—and so in this sense contributed a deductive component—we also invited participants to offer additional comments not grounded in innovation diffusion theory. When we undertook our thematic analysis, we set the theoretical framing “aside” and conducted our thematic analysis inductively such that the themes emerged from the data. Methodological rigor was ensured through comparison and discussion of emerging categories identified independently by two or more researchers and through the use of categories and themes that were robust and supported by data from an array of participants. Data collection and analysis continued until saturation was achieved, i.e., no new insights or themes emerged from our analysis [28]. NVivo10© software was used to manage the data.

## Study results

Four key themes, with embedded sub-themes, emerged from our analysis of the interview data: (1) opinion leader and boundary spanner characteristics; (2) characterizing advice seeking relationships; (3) motivations for providing and seeking advice; and (4) the nature of advice given or sought.

### Opinion leader and boundary spanner characteristics

#### Opinion leader characteristics

**Fig. 2:**
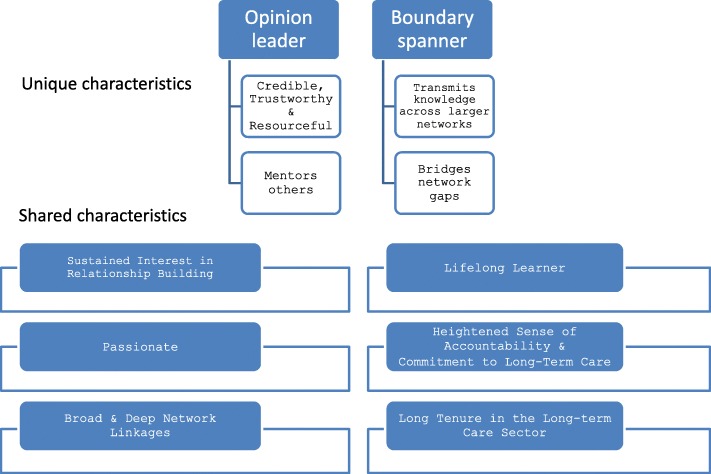
Opinion leader and boundary spanner characteristics

Advice seekers consistently described opinion leaders as well-connected with broad and deep network linkages, diligent in maintaining their connections over their career trajectories, and continuously building more linkages (Fig. 2). Opinion leaders had other attributes that attracted advice seekers: they were seen as reliable, credible, and trustworthy, with reputations as action-oriented, conscientious, approachable, forthcoming, knowledgeable, and willing to share. One advice seeker noted:

It’s based on her expertise. You just know that she knows, and if she doesn’t know, she’ll find out how to get that information for you. She’s very supportive. I can’t overstate this enough, she’s just very fresh and vibrant. Really plugged in. So it’s that personal characteristic of her, as well, that you want to seek her out. (West/North).

Opinion leaders’ networks were not static, but in a constant state of construction and re-construction. They expressed the need to constantly add to and refresh their stores of knowledge, information, and advice, and so they worked continually to build their networks:

I think it’s valuable to share insights and to share experiences … ideas to grow from … we need to stay on top of best practice. And we need to stay on top of educating ourselves and others, the best we can, to care for them. Move away from old ideas. (Opinion Leader, Atlantic).

While opinion leaders were not asked to describe themselves, some of the experiences that they shared regarding their efforts in their networks showcased the characteristics ascribed to them by advice seekers. Opinion leaders expressed what we came to refer to as a heightened sense of “systemness,” that is, they were interested in—and appeared to feel that it is their responsibility to—help progress the care and operations of the LTC system well beyond their own organization and its more narrow interests. For example, some participants described system planning and developing system-wide policies. Many opinion leaders also had remarkable foresight, and they expressed the importance of mentoring others in their networks in the interests of developing future leaders in LTC.

#### Boundary spanner characteristics

Advice seekers described boundary spanners in a manner similar to that of opinion leaders: they were passionate about improving the LTC sector generally, they were approachable and had a good reputation, they were willing to share knowledge, they were lifelong learners, and they consciously and continually worked to build their network connections (e.g., seeking formal networking opportunities to build and maintain connections by attending conferences and through active membership in organizations/associations). They too were described as having a heightened sense of systemness (Fig. 2). Another notable characteristic was their long tenure both in LTC and their current job, which they acknowledged as key for those seeking advice from them because they understood the LTC system. A key defining characteristic of boundary spanners was that they strengthened the network by bridging gaps in the network and they sought to transmit information broadly across the network, information that otherwise would not get shared among network members.

### Characterizing advice seeking relationships

Building advice seeking relationships had three sub-categories: types of advice seeking relationships, evolution of advice seeking relationships, and outcomes of advice seeking relationships.

#### Types of advice seeking relationships

We found four types of advice seeking relationships: *formal*, *peer-to-peer*, *mentoring*, and *reciprocal*. *Formal* advice seeking relationships resulted from hierarchy relationships (e.g., reporting relationships) and were routine, regular, and structured. Advice seekers identified fewer of these than informal relationships, and frequently, a formal opinion leader-advice seeker relationship evolved over time to an informal one. More often, advice seeking relationships originated from existing, longstanding *peer-to-peer* relationships as a result from working together for several years and continued to be fostered through promotions and changes in job positions over the years. In some instances, advice relationships emerged from *mentoring* roles, whereby someone started in a new role and was mentored by a more senior/experienced individual and this relationship continued over time. While formal and mentoring relationships were both structured (e.g., based on seniority), formal relationships were leadership positions that may or may not have included mentoring roles. Reciprocity is a characteristic that most often applied to peer-to-peer relationships, but also characterized mentoring relationships that were sustained and evolved over time. Initially, many mentoring relationships began as a one-way advice seeking relationship yet over time evolved into *reciprocal* advice seeking relationships:

She [opinion leader] had a background in infection control. So questions … around … what they’re doing as best practice … [were the original focus] … Now … we actually bounce things off each other as well. So it’s not as one-way as four years ago. (Advice Seeker, Atlantic).

#### Evolution of advice seeking relationships

Generally, the type of advice sought or given changed as the (type of) advice seeking relationship evolved. Often, relationships began with seeking operational information based on a critical event such as a difficult situation with residents, families, or staff in which the advice seeker wanted specific information to help them to problem solve. Many of the advice seekers described how advice evolved from urgent problem solving to seeking and exchanging strategic and clinical advice. Frequency of advice sought also changed, with more informal advice seeking as comfort grew and trust built. General orienting knowledge and advice was also sought by those newly entering into LTC as leaders, or those adjusting to a change in organization or location, who needed general knowledge to orient themselves to the LTC sector or their new role. As some advice seekers became more familiar with LTC and established in their new contexts, the advice that they sought shifted to include advice on strategic/operations or clinical matters. Frequency also changed as advice seekers became more established in a role or sector and increased their network breadth to rely less exclusively on the original opinion leader:

[advice seeking] is less frequent … I think the confidence level … has grown to where I don’t find I need to seek the advice as often. If we do connect, oftentimes it’s more of a social nature … But there’s still those times where I would go to her, and I feel comfortable to say that I have no problem phoning her up. (Advice Seeker, West/North).

Direction of advice or information flow changed over time, often beginning as one-way exchanges but generally evolving to two-way (reciprocal) exchanges. Many opinion leaders expected reciprocity from advice seekers: when they gave advice or shared information, they also solicited it to build their stock of knowledge of potential use to others in their network.

#### Outcomes of advice seeking relationships

There were four main outcomes of advice seeking relationships: *problem resolution*, *application of new practice*, *co-learning and knowledge exchange*, and *broadened/strengthened networks*. *Problem resolution* was the most commonly described outcome and was often accompanied by *application of a new practice*—clinical or management/operational. As a result of the trust built between the actors in the advice seeking relationships, these relationships formed a valued conduit for seeking solutions for problems emerging in the system. Often, the ability to problem solve was based on historical knowledge within the organization and knowing how to navigate the LTC system:

Sometimes we would go to her [opinion leader], just to ask, “have you heard of any other homes having these same problems and how did they deal with it?” Because she was involved globally, with all 30 homes – more than likely, she’s heard of an instance where it’s happened. So she was able to give some advice, based on learnings from other homes. (Advice Seeker, West/North).

In other instances, the opinion leader shared unsolicited knowledge of a new practice with advice seekers, i.e., not necessarily in response to the presentation of a problem. These were adopted; seemingly, some of the barriers to innovation adoption were overcome simply because of the trust imbued in the opinion leader on the part of the advice seeker. This trust allowed for more reciprocal learning and collaboration and less protectionism over knowledge. While knowledge exchange and broadening/strengthening networks tended to occur largely at the provincial level (i.e., attending conferences), there was evidence of network leaders drawing on their networks in other provinces. Networks were leveraged to enhance co-learning and knowledge exchange, fostering a less insular perspective on problem resolution:

*…* Over the last 10 years, I think people are getting very creative and – and reaching out, not just in the provinces or the territories themselves, to say, “what are you doing and how can we share?” Like we should not be doing this alone. And there’s so much good stuff going on out there, right? And this is a great opportunity. It’s good to see. (Opinion Leader, West/North).

Networks were strengthened by the conversations which allowed for both building rapport and offering a safe place to discuss difficult situations. Sharing information and offering the opportunity to speak and be heard provided many of the network actors a sense of community where the particularities of LTC were understood among colleagues. The collegiality facilitated informal conversations that supported the formation of new ideas and the confidence to try new approaches.

### Motivations for providing and seeking advice

#### Motivations for providing advice

**Fig. 3:**
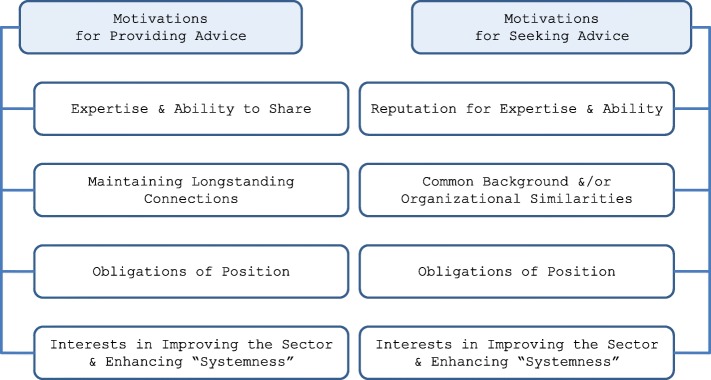
Motivations for providing and seeking feedback

Motivations for providing advice were both altruistic and purposeful for opinion leaders. Generally, they referred to experiencing satisfaction from helping others and sharing their expertise (Fig. 3). They articulated an enduring passion not only in advancing the interests of their organization but to contributing to improving and advancing the LTC system, consistently expressing an enhanced sense of “systemness.”

One opinion leader stated:

I’m very passionate- I’m a really strong leader … my personal interest is in long-term care … I’ve always been a very strong advocate for the best possible care for people- especially with dementia and palliative care in long-term care settings. (West/North).

#### Motivations for seeking advice

Opinion leaders’ reputed expertise motivated the initial contacts made by advice seekers. Similarities in background, experiences, and LTC facility size were also factors that led advice seekers to seek certain opinion leaders:

She’s been recognized as … a good director of nursing … And it’s also a large home … which is important to me, because it’s really hard when you’re almost 200 beds, to be comparing what somebody’s doing in a 30 or 40 bed home … She was approachable … in certain areas, she’s got the answers. She … comes from a strong clinical background … Plus she’s got some good leadership skills. She’s … not afraid to … make hard decisions … (Advice Seeker, Atlantic).

Opinion leaders’ heightened sense of systemness was also a motivator for seeking advice:

… the way she looked at long-term care and thought about long-term care certainly has made me much more comfortable in seeking … her input and her advice in different areas. Because I know how she feels about the sector, you know, her passion for it. And her knowledge of it. (Advice Seeker, Atlantic).

### Nature of advice given or sought

Two types of advice were given and sought: reactive and proactive.

#### Reactive advice

**Fig. 4:**
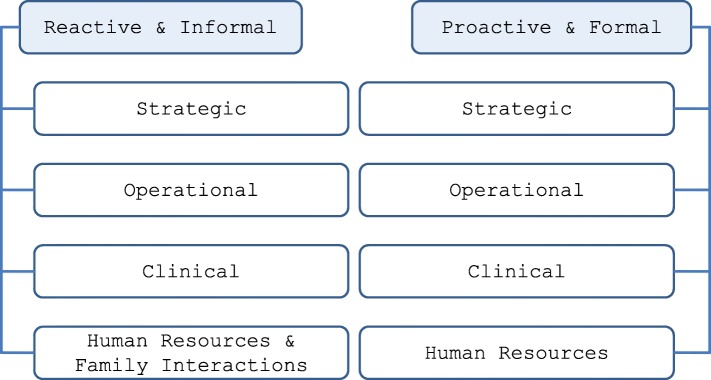
Nature of advice given and sought

Reactive advice was generally transmitted or given informally, when an advice seeker contacted an opinion leader to assist in resolving a problem or help them think through an issue. These problems were generally practical, but they ranged widely, from family concerns/relations to clinical questions to accreditation and regulations to human resource issues (Fig. 4).

#### Proactive advice

Advice seekers acknowledged that it was important to routinely confer with opinion leaders to adhere to standards of care and keep abreast of relevant policies. Proactive advice was generally, and understandably, offered in a formal format, e.g., e-mailed or written communications, in standing committees among Directors of Care or LTC facility administrators. Often, the opinion leaders giving proactive advice were in governance or oversight positions:

I am proactive in the sense that I host regular meetings for all the directors of nursing to attend. And in those meetings, oftentimes our agenda is heavily laden with policies that are coming, that are new, with development related to standards and accreditation that we need to meet. So I think there is that proactive part of it. I also try to, at those meetings, be proactive in bringing some best practices forward. So if - for example, I’ve come across a leading practice related to dementia care, I will try to share that to that team at those meetings, in order to broaden their knowledge base and keep them abreast of whatever developments there are. (Opinion Leader, West/North).

## Discussion

Our study aim was to understand how and why LTC leaders in a pan-Canadian interpersonal network provide and seek advice about care improvement innovations, in order to design more effective dissemination and implementation programs. We discuss our main findings within the context of the extant literature and offer implications for future dissemination strategy and research.

### The dynamic nature of advice seeking relationships

A common thread that ran through each of the four themes identified in our results was the dynamic nature of the advice networks we studied. We observed that (a) the network roles of opinion leader, boundary spanner, and advice seeker can be more complementary and overlapping than traditionally portrayed in the diffusion literature, (b) people can assume different roles in the network over time, and (c) advice relationships can change over time. Most relationships that we examined began as peer-to-peer and evolved over time, solidified by a common appreciation for resident-centeredness and an earnest desire to improve and advance LTC. Reporting or formal authority relationships, in which the flow of advice began as unidirectional, evolved into reciprocal relationships, and advice seekers eventually exchanged knowledge and information with opinion leaders.

This finding both echoes and expands upon the results of previous theoretical and empirical research. Scholars studying advice relationships in professional networks have observed that such relationships are often reciprocal, especially in contexts with less formal hierarchy, where everyone possesses some knowledge of value to network members [29, 30]. In two cross-sectional studies of health care professionals, for example, Keating and colleagues found that reciprocity was a significant predictor of advice ties [31], and Zappa found that reciprocity characterized 93% of such relationships [32]. Our research is consistent with these findings. However, it adds longitudinal insights: although reciprocity has been a much-studied characteristic of network relationships, only a small proportion of studies on professional networks have been longitudinal or have queried relational history [33]. Even in the diffusion literature, which addresses an inherently dynamic process, studies that capture the long-term evolution of advice relationships over professional careers, beyond the diffusion of a single new practice or innovation, are rare. Our finding that among LTC professionals, such advice relationships often become increasingly reciprocal over time and that the initially distinct roles of advice source and seeker eventually blur and overlap represents an expansion in our understanding of the dynamics of roles and reciprocity in advice networks.

### Critical roles in advice seeking networks

In a second main finding from our results, the opinion leader characteristics, described by advice seekers, highlighted the impact that the former have in supporting the interpersonal advice seeking networks to improve the LTC sector. Opinion leaders in our study shared many characteristics identified in previous social network studies: they were perceived as influential, trustworthy, and credible [12, 34, 35]; they were a near-peer friend; [36] and they were accessible [8, 35]. They were also inherently relational and keen to mentor—skills that are important to sustain and strengthen advice networks. However, we observed additional attributes: opinion leaders were described—and *self*-described—as sharing a strong sense of systemness and as being motivated by an interest in advancing LTC generally.

Prior research on opinion leaders offers related, but not identical, observations. For example, in his review of the diffusion of innovations, Rogers noted that opinion leaders are often more “cosmopolitan” than their colleagues, with greater exposure to the bigger picture of advances in their profession beyond the focal advice network, and in some studies have displayed a strong sense of “altruism” toward fellow network members [1]. Studies of collective action networks and why people contribute to public goods—such as quality improvement in the LTC system—have observed that people with more connections in a communication network, such as opinion leaders, are more likely to support the “collective good” in that network [37]. Scholars studying organizational behavior have observed associations between active participation in a professional network and performing “interpersonal citizenship behavior” in that network [38]. The characteristics of cosmopolitanism, altruism, support for the collective good, and interpersonal citizenship behavior are derived from differing empirical contexts and research literature but describe attitudes and behaviors similar to, and perhaps overlapping with, a person’s sense of “systemness,” i.e., a feeling of responsibility to help advance a sector beyond one’s own organization and its more narrow interests. Our observation that a key trait of opinion leaders in our study was their “sense of systemness” contributes to the disparate yet related findings in the literature and suggests that future research on opinion leaders in professional advice networks might usefully pursue confirmation and integration of these concepts.

Consistent with characterizations in other studies [13, 25, 39], we observed that boundary spanners in Canadian LTC networks communicated advice that might well otherwise be stuck within sub-networks. In the social network theory literature, boundary spanners are important conduits of heterogeneous knowledge and advice between groups in networks or from network to network: innovations that originate in one group and might never be shared or transmitted more broadly across the larger network were it not for boundary spanners [13]. Boundary spanners in our study were key to strengthening the network by bridging network gaps, and they actively built their connections over their long tenures in LTC, acquiring a keen understanding of changes in the sector over time. Their relationships with diverse groups of people and their historical knowledge of the network [40] suggest that boundary spanners are likely valuable informants in designing effective dissemination strategies and identifying deficiencies in an existing network (e.g., unrealized ties) as well as playing critical roles to moving innovations from group to group, company to company, province to province.

### The importance of reactive problem-solving in the diffusion process

A third main finding from our results was that informal, reactive problem-solving played a major role in driving advice relationships, and thus best practice dissemination, in the LTC sector. Our participants identified problem resolution as the most common outcome of advice relationships and pointed to reactive advice—advice about a best practice that directly responded to a specific problem articulated by the advice seeker—as one of the two forms of advice exchanged in the network. Informal, reactive problem-solving stands in contrast to the proactive sharing of best practices, often in formal, didactic settings. While our participants reported that the latter form of advice exchange also occurred regularly, our results highlight the need for dissemination planners to recognize that the diffusion process will always likely be driven by a combination of “push” and “pull,” proactive and reactive forces [3]. To expect that a best practice can be effectively disseminated entirely through formal, proactive communication of the practice is unrealistic. As our network members reported, best practices are often offered by an opinion leader as a potential solution to an advice seeker’s problem. This finding supports similar observations by scholars who have applied complexity theory and systems theory to dissemination interventions. This line of research emphasizes a collaborative, negotiated approach tailored to local problems, as opposed to top-down replication of the best practice, allowing for local sensemaking, self-organizing, and adapting of the practice to different contexts during the dissemination and implementation process [5, 41]. For example, a study of whole systems change in health care found that when opinion leaders perceived and communicated a new practice as a potential solution to multiple problems experienced locally throughout an organization or system, wide-scale change was more likely to occur [42].

### Implications for the dissemination of innovations

Our findings suggest several practical implications for future dissemination initiatives. First, those involved in planning dissemination strategies for care improvement in the LTC sector should consider identifying an existing professional advice network and then its opinion leaders, recruiting them to assist with dissemination initiatives. Our findings suggest that organizers of dissemination initiatives can offer to partner with opinion leaders at the planning as well as execution stages. For planning, opinion leaders are likely excellent sources for identifying receptive actors or sites with which to implement an innovation and for framing the innovation so that it is relevant to the specific problems and opportunities faced by those actors and sites [3]. At the execution stage, because opinion leaders actively communicate with advice seekers about innovations, they can be given stock information about a focal practice innovation for dissemination to advice seekers; this is the classic way in which opinion leaders have been utilized [1, 7]. Note, however, that Dearing cautions that dissemination planners should not attempt to co-opt the judgment of opinion leaders: “Opinion leaders are perceived as expert and trustworthy precisely because of their relative objectivity regarding innovations. Indeed, most of their judgments about innovations are negative …Innovations perceived as radical are especially likely to be rejected by opinion leaders and, thus, are better targeted first to innovators who are sources of information for the opinion leaders” ([8] p. 514).

Second, when the goal is not to disseminate a specific practice, but rather to support and prime a network for future dissemination initiatives, opinion leaders in the existing interpersonal advice network can also be recruited to aid intervention planners. Our results indicated that opinion leaders were good sources for identifying the next generation of opinion leaders, possibly from among highly active and experienced advice seekers. In doing so, they can perform a form of network intervention Valente refers to as “alteration” (e.g., adding or deleting network members) [7], but they can do so from within the network, rather than an external program planner trying to impose a change upon the network. Our results also indicated that opinion leaders can contribute to initiatives that enhance systemness, including socializing network newcomers to the system. In these two ways, opinion leaders can help to develop and maintain a network’s capacity for innovation diffusion over time.

Third, knowing that networks and their members constantly evolve, plans can be made accordingly. Opinion leaders and other key actors in a network may change formal hierarchical positions or informal network roles, or retire, disrupting the network and dissemination planning. However, the finding that network roles evolve implies that opinion leadership and boundary spanning are not inherent or immutable personality traits, but rather are behaviors that can be supported and encouraged, particularly with the engagement of a network’s active advice seekers.

Lastly, structures and opportunities should be developed to support opinion leaders to disseminate effective practices in the LTC sector. In comparison to many US states, Canada’s LTC care system is publically regulated and many of the opinion leaders in our study were public employees tasked with insuring that effective practices were disseminated. Other opinion leaders, perhaps similar to the US governance model, were managers of clinical care for head office corporations. They too had a professional role to ensure dissemination of best practices. Efforts should be made to ensure that managers of these complex systems have, at minimum, support for communication and networking activities. The opinion leaders’ passion to improve quality of care for LTC residents and improve quality of the work environment (i.e., systemness) could be harnessed by LTC facilities, corporations, or regulatory entities by deliberately developing structures and opportunities to enable and support opinion leaders to undertake dissemination. These supportive structures and opportunities need not be costly or time-intensive.

### Implications for research

Few studies have followed the evolution of a professional advice network in a particular sector over time. Future research should further explore the nature of complementary and fluid network roles. Based on the results of the present study, we hypothesize that active advice seekers in the network can become opinion leaders over time. This finding needs additional confirmation and elaboration of specific boundary conditions. Future research should further explore whether or not there are differences in how evidence is exchanged based on its source, i.e., research evidence compared to evidence based on experience. Further research could also include a comparison of respondents and non-respondents and discuss their attributes, network position, and drawbacks to network participation. Future studies should also use mixed methods designs which allow for a more complete understanding of professional advice seeking relationships and network structures. A major implication from this study is that opinion leaders and boundary spanners both can serve as on-ramps to best practice pathways for diffusion.

#### Limitations

As in most network studies that collect data on relationships via survey self-report, there is potential for response bias. Individuals who responded to our survey and participated in qualitative interviews may be more enthusiastic and engaged about quality improvement innovations and more connected within the advice network than those who did not respond. Our study is therefore not well equipped to address questions about barriers or drawbacks to network participation.

## Conclusions

The LTC sector in Canada has been marked as lacking continuity between agencies and organizations that operate as disconnected silos [20]. This study offers a different perspective: senior leaders in LTC use informal provincial networks to actively share best practices across the boundaries of geography, job title, organizational affiliation, and seniority. Members share a strong sense of systemness and a common value of providing the best care across the sector. New knowledge about the distinct roles that network actors play in dissemination offers policy-makers a set of insights that can be used for future dissemination efforts.

## Abbreviations

LTC: Long-term care

## Footnotes

1 Note that the formula for the betweenness centrality metric is such that in order to have a high score relative to others in the network, an actor must have at least one outgoing and one incoming tie from other actors (i.e., be both an advice seeker and an advice source). For this reason, all boundary spanners in our study were survey respondents, and thus, all held the professional role of senior leader in an LTC facility.

2 All survey respondents were considered advice seekers because they had at least one outgoing tie (e.g., Directors of Care, Directors of Nursing).
